# Recruitment and Differential Firing Patterns of Single Units During Conditioning to a Tone in a Mute Locked-In Human

**DOI:** 10.3389/fnhum.2022.864983

**Published:** 2022-09-21

**Authors:** Philip Kennedy, Andre J. Cervantes

**Affiliations:** ^1^Neural Signals, Inc., Duluth, GA, United States; ^2^Belize International Institute of Neuroscience, Belize City, Belize

**Keywords:** human brain implantation, neurotrophic electrode, single units, conditioning, recruitment, speech motor cortex, speech prosthetic

## Abstract

Single units that are not related to the desired task can become related to the task by conditioning their firing rates. We theorized that, during conditioning of firing rates to a tone, (a) unrelated single units would be recruited to the task; (b) the recruitment would depend on the phase of the task; (c) tones of different frequencies would produce different patterns of single unit recruitment. In our mute locked-in participant, we conditioned single units using tones of different frequencies emitted from a tone generator. The conditioning task had three phases: Listen to the tone for 20 s, then silently sing the tone for 10 s, with a prior control period of resting for 10 s. Twenty single units were recorded simultaneously while feedback of one of the twenty single units was made audible to the mute locked-in participant. The results indicate that (a) some of the non-audible single units were recruited during conditioning, (b) some were recruited differentially depending on the phase of the paradigm (listen, rest, or silent sing), and (c) single unit firing patterns were specific for different tone frequencies such that the tone could be recognized from the pattern of single unit firings. These data are important when conditioning single unit firings in brain-computer interfacing tasks because they provide evidence that increased numbers of previously unrelated single units can be incorporated into the task. This incorporation expands the bandwidth of the recorded single unit population and thus enhances the brain-computer interface. This is the first report of conditioning of single unit firings in a human participant with a brain to computer implant.

## Background

Conditioning in mammals was first described by Fetz and Baker (1973) who trained monkeys using juice reward to modifying the firing rates of single units (SUs). Monkeys succeeded in increasing the firing rate of one SU and simultaneously decreasing the firing rate of a separate SU recorded through the same electrode. In more recent years, monkeys were trained to increase or decrease firing rates of single units by operant conditioning, sometimes over several days (Moritz and Fetz, 2011). This important result was taken a step further by developing a bi-directional brain computer interface, whereby the conditioning was promoted by electrical stimulation of neurons in surrounding cortex (Fetz, 2015). A “Neurochip” was developed that provides recording and stimulation in a closed loop system that enhances conditioning (Shupe et al., 2021). Additional studies in monkeys report increased firing using operant conditioning and cortical stimulation via the Neurochip (Eaton et al., 2017).

Operant conditioning of SUs in animals has been performed in hippocampus in rabbits (Berger et al., 1983), visual cortex in cats (Shinkman et al., 1974), motor cortex in rats (Arduin et al., 2014), cerebellum in rabbits (Katz and Steinmetz, 1997), parietal cortex in monkeys (Steege et al., 1982), H-reflex in rats (Carp et al., 2001) and globus pallidus in rabbits (Richardson and Thompson, 1985), among other reports. Operant conditioning in human studies have been restricted to EEG and its various bandwidths (Rockstroh et al., 1984). The H-reflex has also been conditioned in humans (Shindo et al., 1994). As the above brief review illustrates, however, conditioning of available SUs in animals or humans can increase or decrease firing rates of SUs, depending on the task. Until now, there are no known reports of conditioning the interface between human single unit firings and computers or machines.

The question addressed in this report seeks to understand the SU firing mechanisms that underlie conditioning in humans. During a decade long study in participant 5 (FDA IDE G960032S), there was an opportunity to study conditioning in a silent singing task. The participant was presented with an audible tone for 20 s, then attempted to silently sing the tone in his head for 10 s, with a prior resting control period for 10 s. Twenty single units were recorded simultaneously while the tone was attached to one single unit, so that he received feedback of the tone every time the SU fired. We theorized that during this task (a) some of the inaudible SUs would be recruited during conditioning, (b) that the recruitment would be task specific, and (c) that different tones would recruit different patterns of SU activity. The data presented here supports these theoretical conjectures.

The importance of these findings plays directly into attempts to provide a high bandwidth interface especially in the face of few recorded SUs. The current emphasis in research on brain-computer interfaces is the simultaneous recording of many hundreds, if not thousands, of neural firings of multi-units (Schwartz, 2004; Ajiboye et al., 2017; Musk and Neuralink, 2019). With conditioning, and the underlying recruitment of more SUs as reported here, the number of SUs can be more modest, though the actual number required in a specific task is still undefined.

## Materials and Methods

### Electrode

**FIGURE 1:**
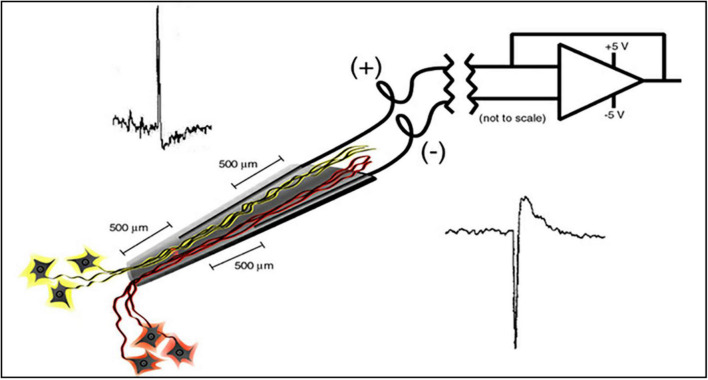
Schematic of the neurotrophic electrode illustrates a hollow glass tip with 2 mil Teflon insulated gold wires glued inside. Trophic factors inside the glass tip entice neurites to grow inside as illustrated. Bipolar recording amplifier produces action potentials that depolarize according to the proximity of the recording wires.

The electrode assembly is shown in Figure 1. Construction has been detailed by Bartels et al. (2008). The cone is made by pulling a heated pipette to produce tip dimensions of 1.5 mm in length, 25 microns at the deep end and a few hundred microns at the upper end to allow space for the inserted wires. 2 mil Teflon insulated gold wires are coiled around a pipette and glued with methyl-methacrylate inside the glass cone. The other end of each coiled gold wire is soldered into a connector that plugs into the implanted electronic component. The electrode is FDA approved (IDE G960032).

### Implanted Electronics

The single channel electronics are assembled in-house and FDA approved (IDE G960032). Bipolar amplifiers record pairs of wires via the low impedance (50–500 kohms) gold wires that are cut across the tips to provide the low impedances. These connect to an amplifier with a gain of 100x and the signals are filtered between 5 and 5,000 Hz. The signals then feed into an FM transmitter operating in the carrier range of 35–55 MHz. During recording sessions, a power induction coil powers the device with the induced current passing through a regulator to provide a stable ± 3 V. The electronics is insulated with polymer (Elvax: Ethylene Vinyl Acetate Copolymer Resin, from DuPont, Wilmington Delaware 1998) and further insulated (and protected against trauma during handling while receiving nursing care) with Silastic (Med-6607, NuSil, Silicone Technology, Carpinteria, CA, United States). The gold pin connection to the electrodes is protected with acrylic cement (Medtronic Inc., Saint Paul, MN, United States). After implantation, the device is covered with scalp skin.

### Implantation Target Site

Because the speech prosthetic is based on movement of the articulators, the speech motor cortex is targeted for implantation. Functional MRI studies during audible and silent speech confirmed the target location as previously described in detail in Bartels et al. (2008). This area extends from the Sylvian fissure medially for 30 mm and about 20 mm in the rostro-caudal dimension.

### Surgery

Briefly, under fully sterile conditions, a craniotomy is performed over the left sided speech motor cortex (patient was right handed) and electrodes are implanted as previously described by Bartels et al. (2008). The electronics are attached in participant 5 and at a later surgery in the speaking human. Recordings begin at month 4 in both participants.

### Recording

The recording systems are detailed elsewhere (Bartels et al., 2008). Briefly, the power induction coil is placed over the scalp and the underlying receiving coil, and powers the implanted electronics. Data from the FM transmitters are received via a coil placed on the scalp over the transmitting coil. These data are sent to a receiver that sends it on to the Neuralynx (Bozeman, MT, United States) computer that contains the Cheetah cluster cutting paradigm that separates the single units from the continuous data stream. The single units are then routed to another computer that drives the paradigm.

### Paradigms

**FIGURE 2:**
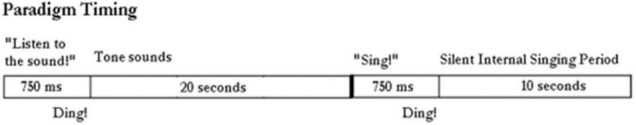
Paradigm timing: The computer outputs “listen to the sound” followed by the tone being emitted from the generator for 20 s. The computer instructs: “sing.” And the subject sings silently in his head. The control period is a rest period in between these actions.

The paradigm used for locked-in participant ER is shown in Figure 2. It consists of the computer first saying “Listen to the sound” followed by the tone for 20 s. Then the computer says “Sing.” We then assume the participant sings the tone in his head. This is repeated at least ten times. We ask him later to confirm that he did sing the tone by rolling his eyes up, or deny saying it by rolling his eyes down. A 10 s rest period prior or after the listen and sing periods provides control data.

Tones are emitted from a generator as sine waves in these frequencies: 110 Hz [A3 (note A in second octave)], 131 Hz (C3), 220 Hz (A3), 247 Hz (G2), 256 Hz (C3), 440 Hz (A4), 494 Hz (B4), 526 Hz (C4), 880 Hz (A5), and 988 Hz (B5). All single units are involved in this conditioning study, whereby the participant is asked to sing silently in his head as accurately as he could. In the first session no feedback is provided. During the second session feedback of single unit firing is provided as a single brief tone each time the single unit fires. During the final session, feedback of single unit firing is provided *and* the audible *volume* of the feedback is directly related to the firing rate. Firing rates are then measured and plotted for comparison.

### Spike Sorting

**FIGURE 3:**
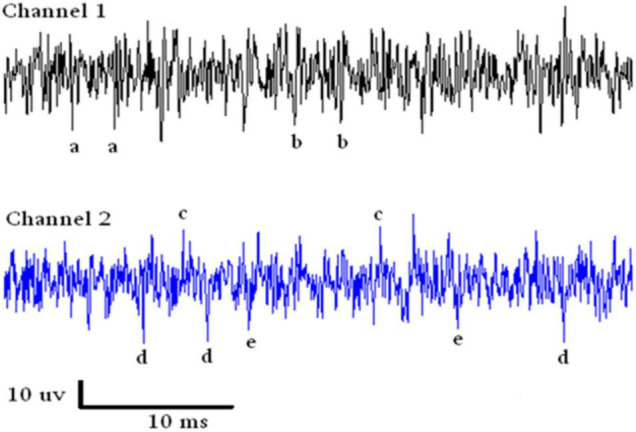
Samples of continuous recording from both electrodes over a 40 ms time base. Due to the configuration of the electrode recording wires, SUs are detectable above and below the continuous stream of data. Thus with voltage level detection there are four channels of data. The lower case letters refer to single units that look similar in amplitude.

**FIGURE 4:**
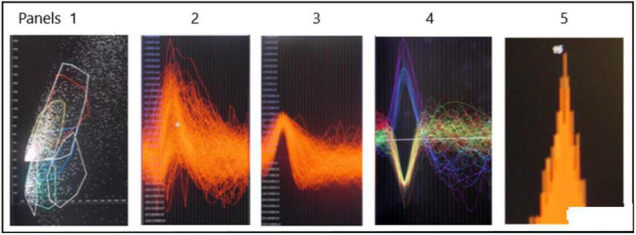
Identification of possible SUs. Panel 1 shows a dot plot of, for example, amplitude versus width of spike. Densities of dots is selected by the user in panel 1 and the many spikes are displayed in panel 2. The putative single spike is separated from other putative spikes by cutting it away using the white marker shown above and below the area of interest. This putative spike is shown in panel 3. Examples of SUs are shown in panel 4. Autocorrelation in panel 5 suggests that the example is likely a SU. The different colors are to distinguish different single units.

**FIGURE 5:**
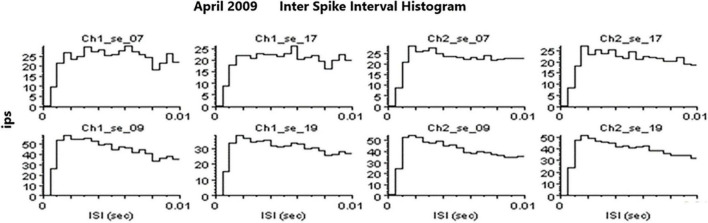
Examples of inter spike interval histograms that identify SUs. Slow firing SU are not shown because they would have a “false gap” due to the slow firing rate.

An example of a continuous stream of neural activity is shown in Figure 3 over a 40 ms time base. Cheetah Spike Sorting software (Neuralynx, Bozeman, MT, United States) is employed to sort the continuous data streams into identifiable single units, of which possible examples can be detected visually in Figure 3 and labeled with letters a, b, c, d, and e. The preferred spike sorting algorithm is the convex hull technique which uses a combination of parameters such as peak, valley, height and area under the curve of the presumptive spikes that are shown in Figure 4. Sampling is at 32K resolution. The program first separates presumed single units using a voltage level set at 11 μVs into upward or downward action potentials thus creating two channels of data for a total of four channels of data. It then applies the parameters for single unit separation to each channel (panel 1). The clusters are selected by circling them with the cursor (as shown in panel 1) to produce multiple waveforms (panel 2). These are then cut or separated by placing a white marker above and below the presumed waveforms which deletes the outlying waveforms resulting in a single waveform (panel 3). This is repeated for many presumed waveforms. This technique is further used to remove extraneous signals from the waveform. Finally, examples of various resultant waveforms are shown in panel 4. Time base is 1 ms in panels 2, 3, and 4. These waveforms are then subjected to auto-correlograms to provide further assurance that they can be designated as single units as evidenced by the single peak (one example in panel 5). Inter spike interval histograms are used to verify fast firing units as single units as evidenced by the 0.5 ms gap at origin (Figure 5). Slow units will have a false gap and are not shown. Further validation of single units is dependent on functional studies as described below.

## Results

**FIGURE 6:**
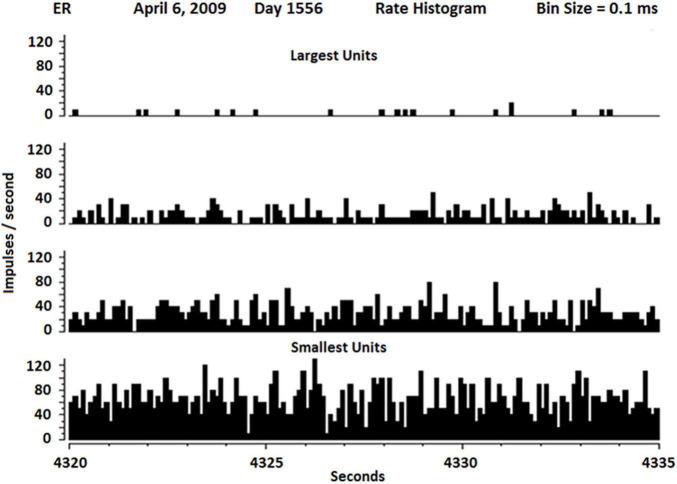
Rate histogram of SU firing over 15 s separate slow (about 1 Hz) to fastest firing SUs (about 100 Hz). The slowest SUs also had the highest amplitude, suggesting they may be Betz cells, whereas the fastest SU had lowest amplitude, suggesting they may be interneurons.

The firing rates of SUs vary widely as shown in Figure 6. Because the neuropil grows in both ends of the glass tip, it is not surprising that Betz cells from layer five and interneurons from layer 2 or 3 would grow into the electrode tip from above (interneurons) and below (Betz). Because interneurons usually have fast firing rates and Betz cells usually have lower firing rates, it is not surprising to see the distribution as shown in Figure 6.

### Recruiting of Single Units During Conditioning

**FIGURE 7:**
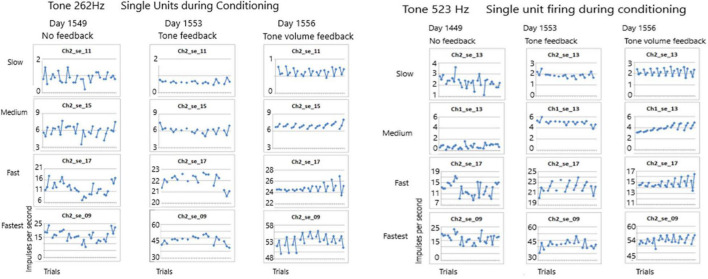
Firing rates of SUs are shown over seven to ten trials for tones of 262 and 523 Hz. Each trial plots the firing rate during listening, resting and silent singing. On day 1549 no feedback was provided during silent singing. On day 1553 feedback of the tone was triggered by firing of SU 2-17. On day 1556, the volume of the tone feedback was increased proportionate to the rate of firing. The conditioning improved across the three sessions.

The first issue addressed in this study seeks to determine if any of these SUs could be conditioned. Conditioning is defined as a non-random firing rate during an active task such as singing. Using the paradigm described above, firing rates were measured during listening to the tone, rest and during silent singing, repeated for 10 trials. As Figure 7 shows, the conditioning of firing rates was random when no feedback was available during silent singing on day 1549 for tones 262 and 523 Hz. A few days later, feedback was provided by triggering the tone for each firing of SU 2-17. This provided some conditioning as can be observed by the symmetry of the firing rates. On day 1556, the volume of the tone feedback was proportionate to the *rate of firing* of the SU 2-17. The SU firing rate conditioning improved and became more symmetrical.

### Task Dependency of Single Unit Recruitment During Conditioning

**FIGURE 8:**
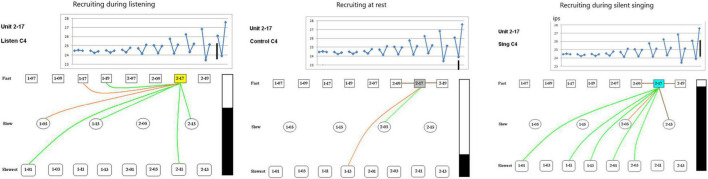
Recruiting of SUs during listening, rest, and silent singing is illustrated as connections between the index SU (#2-17) and fast, slow, or slowest firing units. Green lines indicate increase above baseline firing, brown and red illustrate inhibition of firing to different degrees of inhibition. Tone is C4 (262 Hz). The black bar illustrates the total firing (arbitrary numbers) and is used for comparison between the different tasks. A power point slide in Supplementary Material illustrates trials 4–10.

The second issue examines the possibility of different recruitment patterns during the listen, rest and silent singing phases of the task. As the panels in Figure 8 show, there is indeed a marked difference with enhancement of firing (green) and inhibition of firing (red or brown lines) between the different phases of the tasks. This is illustrated better on a power point slide in Supplementary Material.

### Pattern of Recruitment

**FIGURE 9:**
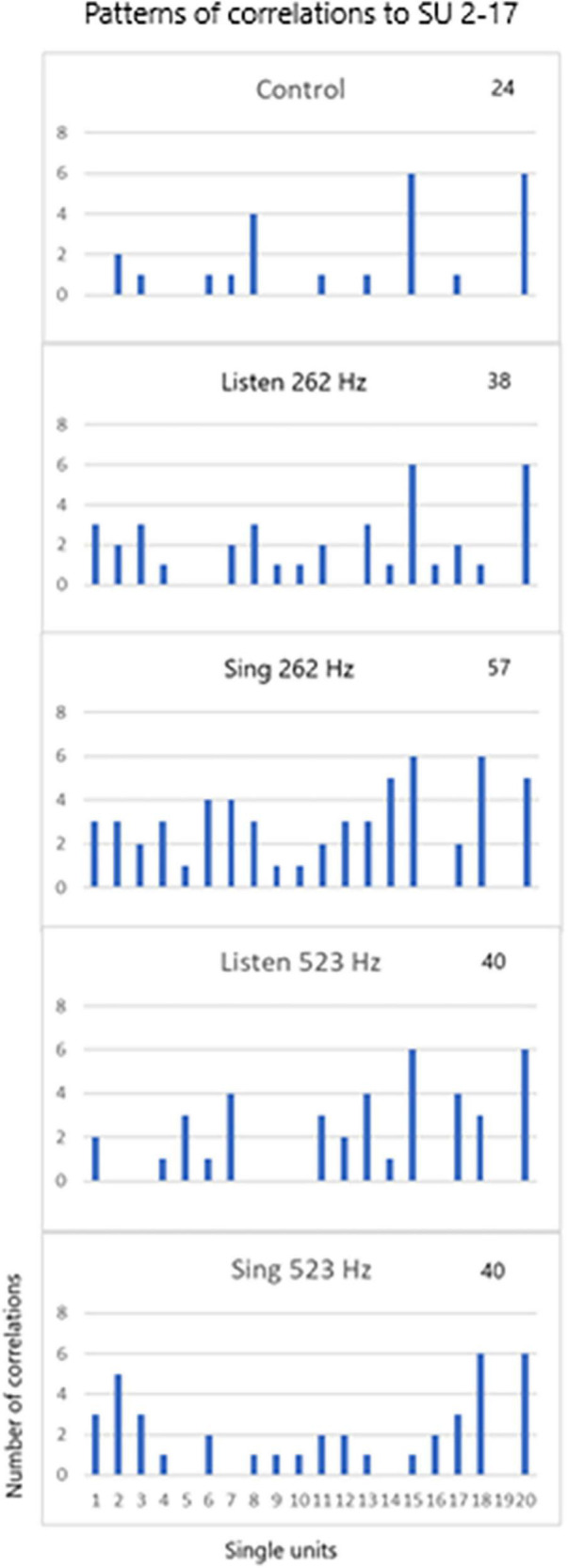
SU 2-17 (SU #19 above) was used to cross correlate (XCorr) with the 19 other units during control periods of resting, periods of listening to the tones (262 and 523 Hz) and silent singing of the tones over six trials. The patterns of XCorrs is different between the different tasks: the pattern identifies the task. The total number of XCorrs (excitatory or inhibitory) is given as a number on each plot. Illustration of the patterns is demonstrated in the power point slide in Supplementary Material.

The third issue deals with the pattern of recruitment during the three tasks for different tones (262 and 523 Hz) for the 20 SUs. Note that SU #17 was used in the cross correlation analysis as shown in Figure 9. The patterns are so distinct that the pattern itself can identify the three tasks for the two different tones.

## Discussion

The results above indicate that some of the non-audible single units were recruited during conditioning as evidenced by the symmetrical firing rates during the three phases (listen, rest, and silent sing) when tone feedback, especially volume feedback, was provided. Importantly, the conditioning improved from one trial to the next as shown in Figure 7. Also, overall firing rates increased as the trials progressed from no feedback to feedback, such that some SUs had a dramatic increase in firing rates after the tone feedback was introduced as can be seen in Figure 7 for both tones. Comparing tone 262 Hz and tone 523 Hz conditioning, there is a sustained “flipping” of firing rate patterns in SU 2-17 as can be seen by the “V” shape of the plotted firing rates. More evidence for identifying the tone from these SU firing patterns is discussed below.

Some SUs were recruited differentially depending on the phase of the paradigm (listen, rest, or silent sing) as shown in Figure 8. Only three examples are shown, but a power point slide in Supplementary Material will show more examples. The lines indicate which SUs had enhanced (green) or inhibited firing (red or brown).

Single unit firing patterns differed between different tone frequencies, 256 versus 523 Hz, as illustrated in Figure 9. It is apparent that the tone could be recognized from the pattern of single unit firing patterns in these examples. In addition, the patterns were specific for the phase of the task (listen, rest, and silent sing), making it likely that the phase could be recognized. Pattern recognition is very important because it is the basis of detecting not just tones, but other modalities such as speech.

**FIGURE 10:**
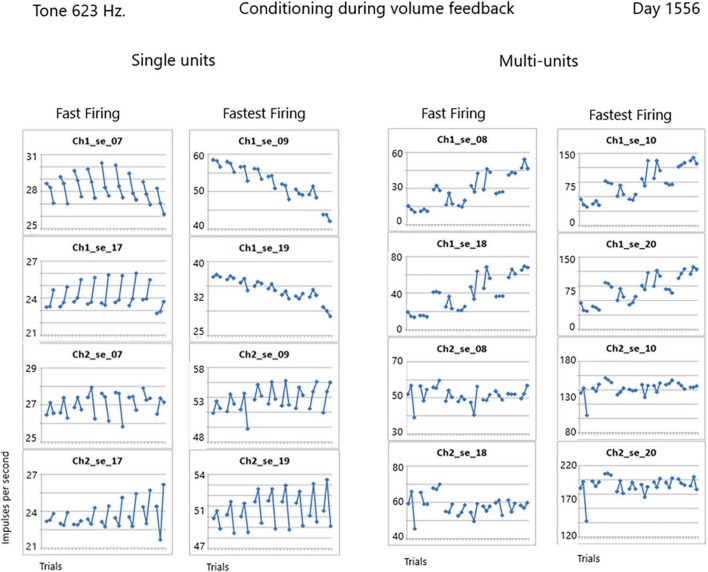
Comparison of single unit and multi-unit firing rates during conditioning with volume feedback recorded on day 1556 after implantation.

No other researchers are known to have published conditioning of human SU data. Instead, other research paradigms use multi-unit activity as a proxy for SU activity. To test the viability of this proxy, we extracted the multi-units from the data along with the SUs on day 1556. Figure 10 illustrates that there is no symmetry to the firing activity of multi-units when compared to the SU data. In other words, the precision available with SU firings is not available with multi-unit activity. Thus this proxy is not valid.

From a practical point of view, multi-units may be adequate for neural prostheses and are being used as such (Hochberg et al., 2012; Homer et al., 2013; Bouton et al., 2016; Ajiboye et al., 2017; Musk and Neuralink, 2019). However, as mentioned above, high bandwidth data will improve precision decoding, and thus improve the quality of speech (or fine finger movement) decoding.

Other workers have used single units with interesting results. Ossmy et al. (2015) recorded human auditory cortex during surgery and detected SU activity related to onset of speech. Studies comparing audible speech and hand use in American Sign Language were studied: Single unit recordings in the anterior temporal lobe, at a site later identified to be important in handshape formation, showed sustained activity during naming with superimposed increases in activity during audible speech (Haglund et al., 1993). No evidence is presented indicating decoding for speech using SUs in these studies.

Other researchers use the frequency domain to decode speech with interesting results. Ramsey’s group use high density ECoG electrode grids to successfully decode phonemes by placing it over the frontal lobe and recording neural activity in the frequency domain (Ramsey et al., 2017). Chang’s group place a grid of ECoG electrodes over the speech areas and reproduce speech using the frequency domain (Conant et al., 2018; Hamilton et al., 2018). More recently, Moses et al. (2021) decoded speech in a locked-in patient using high density ECoG electrodes in real time using data acquired from the gamma band. Decoding was augmented by a natural language model and a Viterbi decoder. Results indicate decoding at 15.2 words per minute with a median error rate of 25.6% (Moses et al., 2021). All these efforts using ECoG recordings are ongoing and may result in natural speech from locked-in people.

Using ECoG recordings, Chang and his group have identified the human lateral primary cortex as the area for control of articulation, in other words, the speech motor cortex (Bouchard et al., 2013). In our studies, this area has been confirmed by recording SUs and detecting phones, words and phrases Brumberg et al. (2011). Hence, implanting the motor speech cortex to restore speech in those locked-in people who have an intact cortex is essentially a motor, not a speech, decoding task (Kennedy et al., 2011).

## Conclusion

The present data indicate that SUs can be conditioned to the task. Though the firing rates were initially random, they began to fire at similar rates as audible feedback was introduced. It is important that the feedback was audible as conditioning occurred only when one SU fired while the other 19 SUs became conditioned as the trials progressed. Thus even though the SUs were not conditioned to the task initially, the SUs became conditioned with feedback. Conditioning with feedback is an important capability of SUs because they expand the bandwidth of the population of recorded neurons and thus enhance the brain to machine/computer interface. It is important to recall that multi-units are not a proxy for SUs in this task but they are used successfully in brain to computer interfaces. The pattern of SU firing is one of the key aspects of decoding and forms the basis of speech decoding (Brumberg et al., 2011).

## Data Availability Statement

The raw data supporting the conclusions of this article will be made available by the authors, without undue reservation.

## Ethics Statement

The studies involving human participants were reviewed and approved by the Neural Signals, Inc., Ethics Committee. The patients/participants provided their written informed consent to participate in this study.

## Author Contributions

PK performed the experiment and analyzed the data. AC performed the surgeries. Both authors contributed to the article and approved the submitted version.

## Conflict of Interest

PK has 98% ownership of Neural Signals, Inc. The remaining author declares that the research was conducted in the absence of any commercial or financial relationships that could be construed as a potential conflict of interest.

## Publisher’s Note

All claims expressed in this article are solely those of the authors and do not necessarily represent those of their affiliated organizations, or those of the publisher, the editors and the reviewers. Any product that may be evaluated in this article, or claim that may be made by its manufacturer, is not guaranteed or endorsed by the publisher.
