# Supplementary material for: Recruitment and Differential Firing Patterns of Single Units During Conditioning to a Tone in a Mute Locked-In Human
Source: Front Hum Neurosci. 2022 Sep 21;16:864983. doi: 10.3389/fnhum.2022.864983 (PMC9532552; doi:10.3389/fnhum.2022.864983)
Supplement: Supplementary file 1 [file Presentation_1.PPTX]

## Slide 1
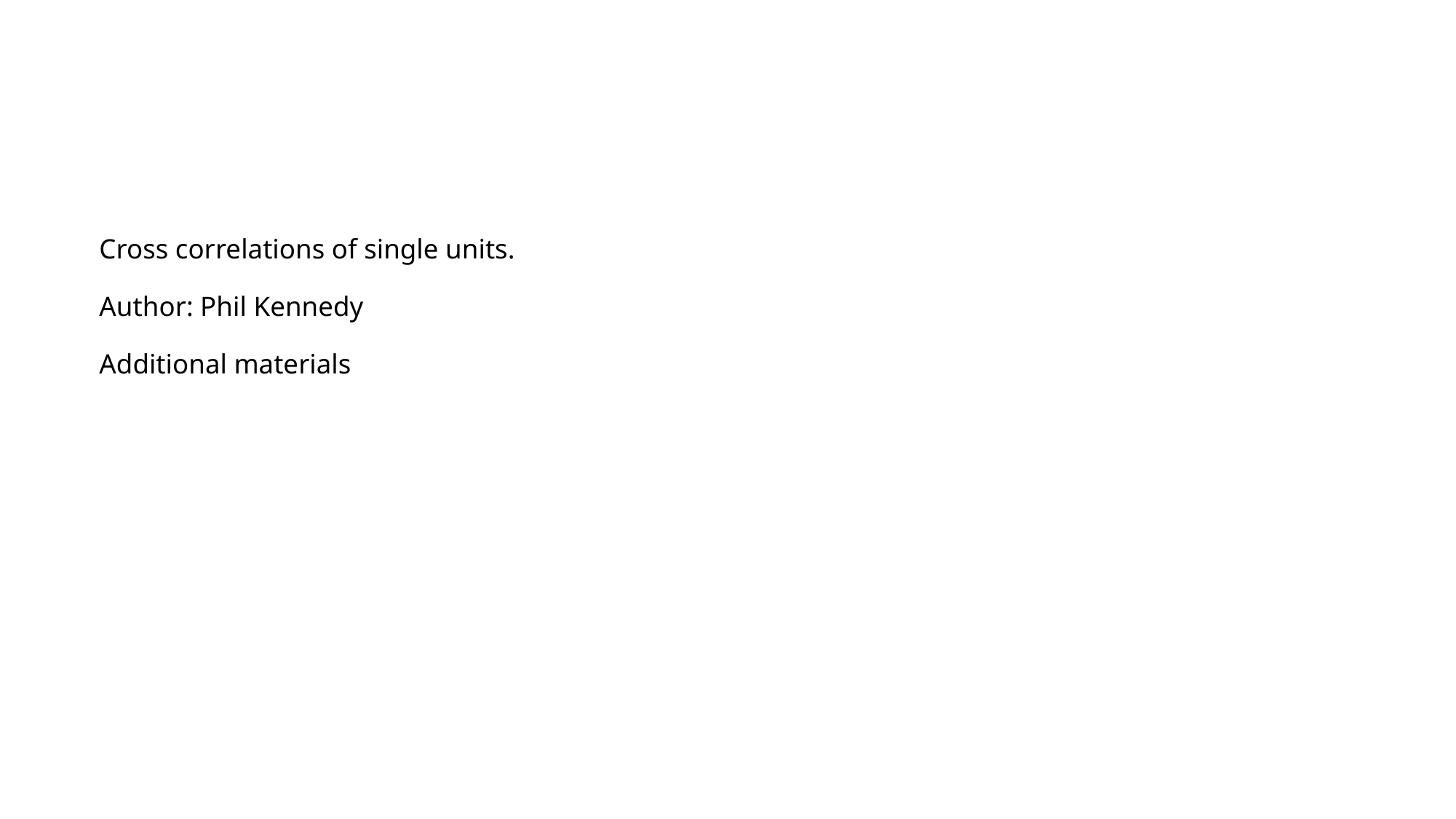

# Cross correlations of single units. Author: Phil Kennedy Additional materials

## Slide 2
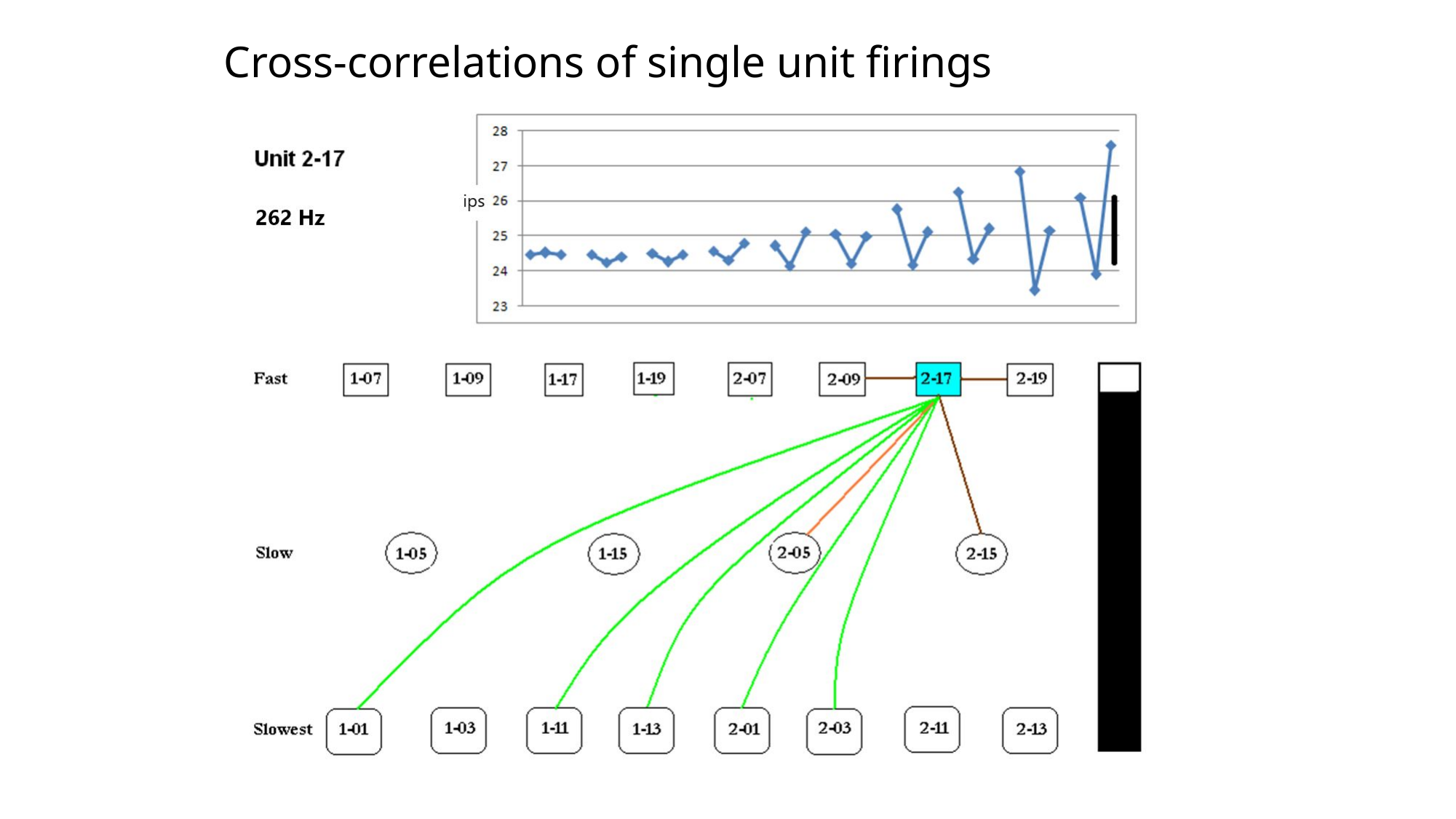

# Cross-correlations of single unit firings
2-17 Correlations
C4 Day 1556
Listen – Control - Sing

## Slide 3
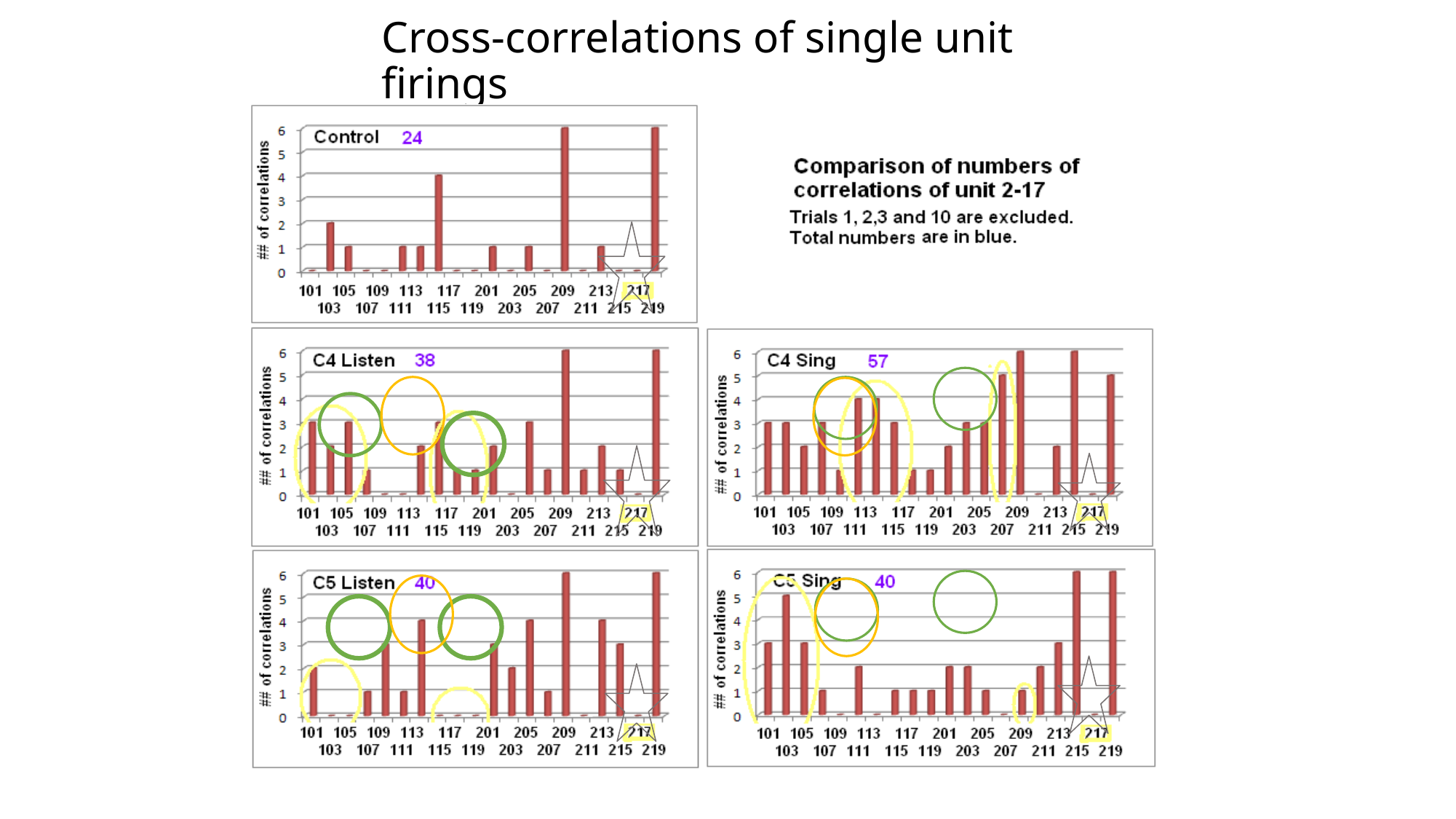

# Cross-correlations of single unit firings
